# Neighborhood socioeconomic status and length of stay after congenital heart disease surgery

**DOI:** 10.3389/fped.2023.1167064

**Published:** 2023-07-18

**Authors:** Sudhir Vashist, Brandon S. Dudeck, Beth Sherfy, Geoffrey L. Rosenthal, Alicia H. Chaves

**Affiliations:** ^1^ Department of Pediatrics, University of Maryland School of Medicine, Baltimore, MD, United States; ^2^ University of Maryland Medical Center, Baltimore, MD, United States

**Keywords:** length of stay, congenital heart disease, surgery, pediatrics, socioeconomic status

## Abstract

**Background and Objectives:**

Socioeconomic factors are associated with health outcomes and can affect postoperative length of stay after congenital heart disease (CHD) surgery. The hypothesis of this study is that patients from neighborhoods with a disadvantaged socioeconomic status (SES) have a prolonged length of hospital stay after CHD surgery.

**Methods:**

Pre- and postoperative data were collected on patients who underwent CHD surgery at the University of Maryland Medical Center between 2011 and 2019. A neighborhood SES score was calculated for each patient using data from the United States Census Bureau and patients were grouped by high vs. low SES neighborhoods. The difference of patient length of stay (LOS) from the Society for Thoracic Surgeons median LOS for that surgery was the primary outcome measure. Linear regression was performed to examine the association between the difference from the median LOS and SES, as well as other third variables.

**Results:**

The difference from the median LOS was −4.8 vs. −2.2 days in high vs. low SES groups (*p* = 0.003). SES category was a significant predictor of LOS in unadjusted and adjusted regression analyses. There was a significant interaction between Norwood operation and SES—patients with a low neighborhood SES who underwent Norwood operation had a longer LOS, but there was no difference in LOS by SES in patients who underwent other operations.

**Conclusions:**

Neighborhood SES is a significant predictor of the LOS after congenital heart disease surgery. This effect was seen primarily in patients undergoing Norwood operation.

## Introduction

Socioeconomic factors at the individual and neighborhood levels affect the health of children and adults in the United States. Lower socioeconomic status (SES) neighborhood scores in Baltimore, Maryland, are associated with a greater all-cause mortality in adults when compared with the known effects of sex, race, and individual poverty status (1). Living in disadvantaged neighborhoods increases the risk for coronary events in adults, even if race, personal economic factors, and medical risk factors are taken into account (2). Similarly, in communities with lower income, the mortality in children with complex chronic conditions is higher (3). Outcomes for children with congenital heart disease are also affected by socioeconomic factors. Children with government-sponsored insurance are at increased risk for mortality, readmission, and prolonged length of stay (4–9). Lower neighborhood SES is also associated with increased mortality (10, 11), readmission rates (12), and length of stay (8, 11) after congenital heart disease (CHD) surgery.

Race also affects healthcare outcomes. However, the interaction between race, ethnicity, and SES is complex (13). Black males with family income below the poverty level have been shown to have the overall highest mortality compared with other demographic groups, but mortality is even lower if they live in a neighborhood of higher SES (1). Asthma readmission rates are higher in Black children and in children living in areas of poverty (14). Similarly, race is a variable associated with outcomes in CHD patients. Non-White patients experience a lapse in pediatric cardiac care at a younger age than White patients (15). Black and Hispanic children with CHD have an increased risk for postoperative mortality, as shown in most studies (16–20), although some studies show no effect of race on mortality (4, 8). Black race and low neighborhood income are risk factors for prolonged length of stay in neonates after CHD surgery (8). Bucholz et al. (10) found an interaction between race and neighborhood SES, with White patients from neighborhoods of high SES having a borderline survival advantage after the Norwood procedure over those from lower SES neighborhoods, while there was no difference in mortality by neighborhood SES in Black patients. Studies, to date, have analyzed the effect of neighborhood socioeconomic factors on readmission rates and length of stay in specific high-risk CHD patient populations (10, 12), and have been performed in a healthcare system different from the US system (21), or have used solely median zip code income as the neighborhood economic indicator (5, 7, 11). The hypothesis of this study is that pediatric patients from neighborhoods with a disadvantaged SES have a prolonged length of hospital stay after CHD surgery.

## Methods

This study was a single-center, retrospective chart review of patients who received care at the University of Maryland Medical Center Children's Hospital in Baltimore, Maryland, an urban tertiary medical center, following CHD surgery. The University of Maryland Baltimore Institutional Review Board approved the study. A total of 282 benchmark procedures, as defined by the Society for Thoracic Surgeons (STS), were performed on pediatric patients aged from birth to 18 years of age between 11 June 2011 and 31 December 2019. If the patient underwent multiple benchmark surgeries during the study period, the first surgery was used for analysis. Patients were excluded if they died before discharge or if an address at the time of surgery was not available. Patient demographic and surgical data were previously collected at our center for submission to the STS Congenital Heart Surgery Database. From the STS database, we collected the following data: sex, preoperative respiratory failure or insufficiency, chromosomal abnormality, race/ethnicity (as identified by the patient or parent, STS uses the categories for race—Caucasian, Black/African-American, Asian, American Indian/Alaskan Native, Native Hawaiian/Pacific Islander, or other and ethnicity—non-Hispanic/Latino or Hispanic/Latino), benchmark surgery (arterial switch, arterial switch with ventricular septal defect closure, atrioventricular septal defect repair, coarctation repair, Fontan procedure, Glenn procedure, Norwood procedure, tetralogy of Fallot repair, truncus arteriosus repair, or ventricular septal defect closure), primary language, primary payor, age at surgery, weight at surgery, hospital LOS, and postoperative complications. The address at the time of surgery was verified in the medical record. Additional data, such as inotrope doses, history necrotizing enterocolitis, and need for sedation weaning for withdrawal symptoms, were abstracted from the medical record.

Date of surgery and date of discharge to home were used to calculate the postoperative length of stay. The STS releases data harvest reports every 6 months, which include the database results for the previous 4 years, including median length of stay by benchmark surgery. Ranges for the STS median length of stay by procedure are shown in Table 1. The STS median length of stay for the appropriate benchmark surgery from the harvest closest to the patient's date of surgery was subtracted from the patient's actual length of stay to obtain a difference from the STS median hospital length of stay.

**Table 1:** Range of STS median length of stay (varies for each 6-month harvest).

Benchmark procedure	Median length of stay (days)
Ventricular septal defect repair	7.7–9.2
Tetralogy of Fallot repair	10.2–11.8
Coarctation repair	10.4–14.9
Atrioventricular septal defect repair	14.2–17.9
Arterial switch operation	15.4–17.4
Arterial switch with ventricular septal defect repair	16.1–22
Glenn shunt	12.1–14.5
Fontan	13.3–14
Truncus arteriosus repair	25.4–29.7
Norwood procedure	38.3–49.8

An SES score for each patient was calculated using methods described by Diez et al. (2) The patient's home address at the time of surgery was used to find a Geocode from the United States Census Bureau. This Geocode was then used to obtain six variables for the patient's census block group from the Census Bureau database: median household income (dollars), median home value (dollars), residents with a professional or managerial job (percentage), households receiving income from interest, dividends, or rent (percentage), residents over 25 years of age who completed high school (percentage), and residents over 25 years of age who completed college (percentage). For each category, a Z-score was calculated using our patient population mean and standard deviation. Although previous studies used an SES score that was the sum of the Z-scores, we found that median home value was missing for some census block groups. Since the missing values tended to be from census block groups with lower z-scores for other variables, we decided to use a mean of the z-scores for the SES score rather than excluding those patients. The SES score was dichotomized to greater than or less than zero, with greater than zero being a higher neighborhood SES score.

### Statistical analysis

Data are presented as frequencies for categorical variables and as means or medians for continuous data with either standard deviation or interquartile range (IQR) depending on the normality of the distribution. The difference in the medians test was used to compare the difference from the STS median hospital LOS in patients with an SES score greater than zero and less than zero. Stepwise linear regression was used to examine associations of variables with the difference from the STS median hospital LOS. Variables with a significant association with the difference from the STS median hospital LOS, such as preoperative respiratory problems, reoperation, unplanned cardiac catheterization, need for sedation wean, and inotrope score, were evaluated using linear regression to determine their role as confounders or effect modifiers. Interaction factors were also tested in the predictive model, including the interaction between SES score and Black race, and included in the final model if they were statistically significant. All statistical analyses were performed using IBM SPSS Statistics Version 27 (IBM Corp., Armonk, New York, USA). The results were considered significant if the *p*-value was less than 0.05.

## Results

Data were obtained on 251 patients. Demographic and preoperative patient characteristics are presented in Table 2. Eighty-one patients underwent surgery as a neonate and had a median gestational age of 38 weeks (IQR 38–40) and a median birth weight of 3.2 kg (IQR 2.4–4). The frequencies of postoperative complications are reported in Table 3.

**Table 2:** Demographic and preoperative factors.

Variable	Categories	*N* (%) or median (IQR)
Sex	Female	104 (41.4)
	Male	147 (58.6)
Race/ethnicity	Black	106 (42.2)
	White	98 (39)
	Asian	8 (3.2)
	Pacific Islander	1 (0.4)
	Hispanic	23 (9.2)
	Other	15 (6)
Primary language	English	234 (93.2)
	Spanish	15 (6)
	Other	2 (0.8)
Primary payor	Government	176 (70.1)
	Private	74 (29.5)
	Self-pay	1 (0.4)
Benchmark surgery	Arterial switch	11 (4.4)
	Arterial switch with VSD closure	8 (3.2)
	Atrioventricular septal defect repair	21 (8.4)
	Coarctation repair	36 (14.3)
	Fontan	4 (1.6)
	Glenn	22 (8.8)
	Norwood	33 (13.1)
	Tetralogy of Fallot repair	48 (19)
	Truncus repair	5 (2)
	VSD closure	63 (25.1)
Previous surgeries	1	22 (8.8)
	More than 1	13 (5.2)
Preoperative respiratory failure or insufficiency	Yes	33 (13.1)
	No	218 (86.9)
Chromosomal abnormality	None	196 (78.1)
	Trisomy 21	37 (14.7)
	22q11 deletion	4 (1.6)
	Other	14 (5.6)
Age at surgery (days)		118 (7, 222)
Time between surgery and harvest (days)		107 (40, 178)
Weight at surgery (kg)		5.17 (3.55, 7)
Height at surgery (cm)		57.5 (49.7, 64.5)
Gestational age (weeks, neonates only, *n* = 81)		38 (38, 40)
Birth weight (kg, neonates only, *n* = 81)		3.2 (2.4, 4)

VSD, ventricular septal defect; IQR, interquartile range.

**Table 3:** Frequencies of complications.

Complication	*N* (%)
Reintubation	45 (17.9)
Reoperation	48 (19)
Open chest	34 (13.5)
Cardiac arrest	3 (1.2)
Postop arrhythmia	42 (16.7)
Sedation wean	42 (16.7)
Prolonged intubation	8 (3.2)
Vocal cord dysfunction	13 (5.2)
Diaphragm paralysis	10 (4)
Chylothorax	14 (5.6)
Sepsis	2 (0.8)
Necrotizing enterocolitis	10 (4)
Superficial wound infection	14 (5.6)
Deep wound infection	3 (1.2)
Mediastinitis	1 (0.4)
Wound dehiscence	2 (0.8)
Unplanned catheterization	5 (2)
Discharged to acute or chronic care	54 (21.5)

The difference from the STS median hospital LOS was −2.2 days in patients with a low neighborhood SES score and −4.8 days in patients with a high neighborhood SES score (*p* = 0.003). There was no difference in hospital LOS by racial or ethnic group.

A simple linear regression analysis showed a significant relationship between the difference from the STS median hospital LOS between a high SES score and a low SES score (*β* = 13.99, intercept = 8.169, *p* = 0.019)—patients from low SES score neighborhoods (SES score <0) had a longer hospital length of stay (or a larger positive difference from the STS median LOS) than those from high SES score neighborhoods (SES score >0) (Table 4). Multiple patient, preoperative, and postoperative factors were associated with a longer difference from the STS median LOS (Table 4). Race and ethnicity were not associated with the difference from the STS median hospital LOS. Those variables that were significantly associated with the SES score, and are thus potential confounders, are shown in Table 5.

**Table 4:** Unadjusted model.

s	Beta	*p*-value	Intercept	95% Confidence interval
Age (days)	−0.008	0.036*	17.997	−0.016, −0.001
Sex	−0.58	0.928	15.477	−13.274,12.113
Socioeconomic factors:				
SES neighborhood score <0	13.992	0.027*	8.169	1.608, 16.908
Preferred language other than English	50.835	<0.001*	11.694	26.773, 74.898
Public insurance	14.837	0.032*	4.733	1.302, 28.372
Latino vs. not Latino	5.089	0.667	14.246	−18.268, 28.446
White vs. not White	7.377	0.255	10.699	−5.362, 20.116
Black vs. not Black	0.818	0.898	14.674	−11.797, 13.433
Preoperative and surgical factors:				
Chromosomal abnormality	2.691	0.726	14.547	−12.422, 17.804
Preop respiratory problem	55.722	<0.001*	7.811	38.574, 72.870
Norwood	54.421	<0.001*	7.982	37.208, 71.634
Bypass time (minutes)	0.174	<0.001*	−11.653	0.086, 0.263
STAT category				
1	−51.042	<0.001*	62.403	−70.360, −31.723
2	−56.111	<0.001*		−77.174, −35.047
3	−60.829	<0.001*		−82.731, −38.926
4	−47.165	0.003*		−78.155, −16.174
5 (ref)				
Postoperative factors:				
Any complication	30.630	<0.001*	1.713	18.621, 42.639
Reoperation	52.846	<0.001*	5.031	38.379, 67.313
Reintubation	61.294	<0.001*	4.148	46.899, 75.689
Cardiac arrest	−10.024	0.732	15.257	−67.551, 47.504
Arrhythmia	−0.23	0.978	15.176	−16.982, 16.522
Intubation >7 days	108.341	<0.001*	11.684	75.412,141.27
Necrotizing enterocolitis	100.799	<0.001*	11.121	71.407, 130.19
Vocal cord injury	7.543	0.599	14.746	−20.655, 35.747
Diaphragm paralysis	27.686	0.087	14.034	−4.098, 59.470
Chylothorax	8.796	0.525	14.646	−18.429, 36.022
Sepsis	62.612	0.079	14.638	−7.284, 132.508
Superficial wound infection	26.990	0.05*	13.632	−0.049, 54.028
Deep wound infection	6.676	0.819	15.057	−50.859, 64.211
Mediastinitis	95.443	0.058	14.757	−3.104, 193.990
Wound dehiscence	−18.585	0.603	15.285	−88.878, 21.560
Unplanned cardiac catheterization	47.713	0.035*	14.187	3.359, 92.067
Sedation wean	70.732	<0.0001*	3.301	56.495, 84.969
Inotrope score	2.633	<0.001*	−7.789	1.653, 3.612

*p-value < 0.05.

**Table 5:** Logistic regression analysis results: odds ratios indicate odds of living in neighborhood with the SES score below the mean.

Variable	Odds ratio	*p*-value	95% Confidence interval
Public insurance	5.419	<0.001	2.915, 10.072
White vs. not White	0.554	0.024	0.332, 0.925
Black vs. not Black	3.268	<0.001	1.938, 5.509
Preop respiratory problem	0.456	<0.046	0.211, 0.986
Reoperation	2.366	<0.011	1.222, 4.58
Superficial wound infection	0.154	0.016	0.034, 0.705
Inotrope score	1.046	0.037	1.003, 1.091

Multiple linear regression showed that SES score, Norwood operation, preoperative respiratory insufficiency, reoperation, superficial wound infection, and government insurance were all significant predictors of difference from the STS median hospital LOS (Table 6). All these variables were associated with a longer LOS. A significant interaction was noted between SES score and preoperative respiratory insufficiency, showing that the combination of living in a low SES score neighborhood and having preoperative respiratory insufficiency was associated with an even longer LOS than the added time associated with each variable alone. There was also a significant interaction between the SES score and the Norwood operation, meaning that the effect of a lower neighborhood SES was greater for patients who underwent the Norwood procedure than for those who underwent other procedures. A further stratified analysis of the Norwood and non-Norwood groups, also presented in Table 6, showed that a lower SES score was a predictor of a more positive difference from the STS median hospital LOS in the Norwood group of patients (i.e., in patients after they underwent the Norwood operation), although the *p*-value was only 0.055, but not in the non-Norwood group of patients. Adjusting the model for reoperation and government insurance in the Norwood group showed no further association between the SES score and the difference in the STS median hospital LOS. The SES score was not a predictor of the difference from the STS median hospital LOS in patients who underwent other surgeries. Although race was not significantly associated with the difference from the STS median hospital length of stay, assessment for effect modification of race on SES score did show that, while the SES score did not predict LOS in Black patients, it was a significant predictor for LOS in non-Black patients. The median difference from the STS median LOS for Black patients was −4.2 days for those from a high SES score neighborhood and −3.2 days for those from a low SES score neighborhood (*p* = 0.537). For non-Black patients, it was −5.2 days for those from a high SES neighborhood and −1.4 days for those from a low SES neighborhood (=0.002). The association between the SES score and the difference from the STS median LOS in non-Black patients was not significant when adjusted for the Norwood operation (Table 6).

**Table 6:** Adjusted models.

Predictor	*β*	*p*-value	Intercept	95% CI
Adjusted model without interaction factors, full cohort (*n* = 251)				
Norwood	30.78	<0.001	−3.631	14.186, 47.373
SES	11.84	0.043		0.383, 23.225
Gov. payor	13.597	0.024		1.759, 25.435
Preop resp.	48.007	<0.001		32.097, 63.917
Reoperation	27.514	<0.001		12.416, 42.613
Wound infection	23.876	0.048		0.183, 47.569
Adjusted model with interaction factors, full cohort (*n* = 251)				
Norwood	10.474	0.345	15.083	−11.327, 32.275
SES	3.245	0.599		−8.904, 34.11
Norwood*SES	42.182	0.009		10.654, 73.71
Gov. payor	13.415	0.026		1.639, 25.192
Preop resp.	35.94	<0.001		16.695, 55.184
Preop resp*SES	37.182	0.025		4.68, 69.684
Reoperation	19.707	0.013		4.129, 35.285
Wound infection	26.973	0.026		3.276, 50.67
Unadjusted model, non-Norwood (*n* = 218)				
SES	5.410	0.209	5.302	−3.054, 13.875
Unadjusted and adjusted models, Norwood (*n* = 33)				
SES	67.013	0.055	27.881	−1.62, 135.65
SES	16.311	0.678	76.916	−63.121, 95.742
Gov. payor	46.706	0.207		−27.256, 120.669
Reoperation	67.78	0.121		−18.77, 153.434
Unadjusted model, Black patients (*n* = 106)				
SES	7.968	0.346	9.411	−8.429, 24.366
Unadjusted and adjusted model, non-Black patients (*n* = 145)				
SES	21.004	0.03	7.653	2.027, 39.981
SES	15.024	0.094	0.747	−2.602, 32.650
Norwood	68.293	0.012		15.360, 121.227

SES, socioeconomic score; Gov. payor, government insurance; Preop resp, preoperative respiratory insufficiency; wound infection, superficial wound infection; CI, confidence interval.

Reoperations were done in 48 patients but were more likely done after the Norwood operation (*n* = 20) with an odds ratio of 10.4 (*p* < 0.001) compared with those done after other operations. In some cases, this included patients who stayed in the hospital during the performance of the Norwood and Glenn operations, with Glenn being considered the reoperation.

## Discussion

It is increasingly recognized that socioeconomic and racial disparities impact healthcare delivery and both short- and long-term outcomes in children with CHD (22–26). In this retrospective single institution study, we sought to assess the impact of neighborhood SES score and race on hospital LOS after CHD surgery. We found that CHD surgical patients with a lower neighborhood SES score had a longer hospital LOS when compared with patients with a higher neighborhood SES score. Anderson, et al. (11) previously described similar findings in their retrospective review of the Pediatric Health Information System (PHIS) database. Children from the lowest-income neighborhoods undergoing cardiac surgery had 1.18 times the odds of mortality and 7% longer lengths of stay than children from the highest-income neighborhoods. However, only median annual household income by patient zip code was used as the determinant of SES.

A subanalysis in our patients showed that the neighborhood SES score was primarily a predictor of the difference from the STS median hospital LOS in patients after the Norwood operation. However, it did not quite reach statistical significance (*p* = 0.055), and this may be attributed to the fact that the number of patients in this group did not provide sufficient power. Similar to our study, a retrospective review of the National Pediatric Cardiology Quality Improvement Collaborative data also showed that a low SES (measured by the deprivation index) was associated with an increased LOS following first surgical palliation in patients with hypoplastic left heart syndrome (27). Similar results were seen in other single-center studies (28, 29). In contrast, a study evaluating neighborhood SES and outcomes following Norwood surgery found that a low SES was associated with worse 1-year transplant-free survival but with no significant differences in LOS (10).

Adjusting the model for reoperation in the Norwood group in our study showed no further association between SES and the difference from the STS median hospital LOS. In most cases, the reoperation was the Glenn surgery, and therefore, the patients with the longer LOS included those who stayed in the hospital after the Norwood operation until their Glenn operation. The interstage period is a vulnerable stage in the care of single ventricle patients requiring close surveillance, complex feeding protocols, outpatient medical equipment, and homecare service needs, all of which results in greater resource utilization (29). Some patients require hospitalization during the interstage for medical reasons, but healthcare providers are more likely to keep interstage infants from low socioeconomic and minority backgrounds in the hospital until the performance of the Glenn operation because of real or perceived concerns about lack of family support systems, reduced financial resources, language barriers, transportation challenges, or poor access to home healthcare services. SES has been previously shown to be a predictor of likelihood for discharge in the interstage period (30). An analysis of the PHIS database noted an increased LOS in Black neonates after CHD surgery and suggested that hospitals may develop care pathways that prolong LOS to mitigate risk in socioeconomically disadvantaged patients, which may have resulted in improved survival rates observed in Black neonates in their study (22).

When assessing the impact of race on LOS, this study demonstrated no significant difference from the STS median hospital LOS between Black and non-Black patients. However, when we assessed the effect modification of race on SES, SES was significant predictor for LOS in non-Black patients, with high SES patients having a shorter LOS. Bucholz et al. (10) similarly found that White patients with high SES had a significant survival advantage over White patients with low SES. The same was not true for Black patients in that study either. The lack of returns in health-related outcomes with higher SES in Black patients has been previously observed in adults after acute myocardial infarction and has been attributed to “diminishing return” hypothesis—Black patients with high SES do not always enjoy the health benefits of increased SES seen by White patients (10). Proposed mechanisms are that minorities living in areas with some of their own racial groups may have poorer health, may have less social support, and greater discrimination with stretched financial resources (10).

### Limitations/explanations

The neighborhood SES score used in our study may not be a true indicator of individual-level SES. Lower-income families living in high-income neighborhoods have been shown to have higher mortality (31), and neighborhood SES has also been shown to have effects on health outcomes beyond the SES of individual patients (2). The racial and ethnic composition of our patient population is different from that seen in other centers, which may limit the generalizability of our findings. An additional limitation is that we used the mean of Z-scores of the six variables of the SES because the median home value was missing for some census block groups. This is in contrast to previous studies that used an SES score that was the sum of the Z-scores. We did not verify all the data that were previously entered for our STS submission, and therefore, it is possible that some data are inaccurate. There were also some missing data, although these were limited. We used the first surgery of patients to avoid correlation of outcomes between patients, but this skewed the distribution of surgeries (i.e., more patients who underwent the Norwood surgery were included compared with those who underwent the Glenn/Fontan surgery).

## Conclusion

As advances in medical, surgical, and post-operative care have significantly helped improve the outcomes of children with congenital heart disease, recent focus has shifted to exploring the role of socioeconomic and racial disparities as additional potentially modifiable factors. Our study contributes to a growing body of literature suggesting the role of SES and race in outcomes of patients with CHD. Modifying practices to screen for these disparities, instituting customized multidisciplinary care pathways, and advocating for increased community healthcare resources to fill these gaps can further help improve CHD outcomes. Providers should also take into account their biases with regard to the ability of a family from a disadvantaged socioeconomic background to provide home care.

## Data Availability

The original contributions presented in the study are included in the article/Supplementary Material, and further inquiries can be directed to the corresponding author.
